# Characterization of an H10N8 influenza virus isolated from Dongting lake wetland

**DOI:** 10.1186/1743-422X-8-42

**Published:** 2011-01-27

**Authors:** Hongbo Zhang, Bing Xu, Quanjiao Chen, Jianjun Chen, Ze Chen

**Affiliations:** 1 State Key Laboratory of Virology, Wuhan Institute of Virology, Chinese Academy of Sciences, Wuhan 430071, PR China; 2 College of Life Science, Hunan Normal University, Changsha 410081, Hunan, PR China; 3 Shanghai Institute of Biological Products, Shanghai 200052, PR China; 4 Graduate University of Chinese Academy of Sciences, Beijing 100049, PR China; 5 Department of Environmental Science and Engineering, Tsinghua University, Beijing, 100084, PR China

## Abstract

**Background:**

Wild birds, especially those in wetlands and aquatic environments, are considered to be natural reservoirs of avian influenza viruses. It is accepted that water is an important component in the transmission cycle of avian influenza virus. Monitoring the water at aggregation and breeding sites of migratory waterfowl, mainly wetland, is very important for early detection of avian influenza virus. The epidemiology investigation of avian influenza virus was performed in Dongting lake wetland which is an international important wetland.

**Results:**

An H10N8 influenza virus was isolated from Dongting Lake wetland in 2007. Phylogenetic analysis indicated that the virus was generated by multiple gene segment reassortment. The isolate was lowly pathogenic for chickens. However, it replicated efficiently in the mouse lung without prior adaptation, and the virulence to mice increased rapidly during adaptation in mouse lung. Sequence analysis of the genome of viruses from different passages showed that multiple amino acid changes were involved in the adaptation of the isolates to mice.

**Conclusions:**

The water might be an important component in the transmission cycle of avian influenza virus, and other subtypes of avian influenza viruses (other than H5, H7 and H9) might evolve to pose a potential threat to mammals and even humans.

## Background

All 16 hemagglutinin (HA) and 9 neuraminidase (NA) subtypes of influenza A virus have been isolated from wild birds [1,2]. Therefore, wild birds, especially those in wetlands and aquatic environments, are considered to be natural reservoirs of avian influenza viruses[2]. It is accepted that water is an important component in the transmission cycle of avian influenza virus, because shedding of virus into the water leads to transmission among wild birds and poultry via the indirect fecal-oral route [2,3].

Dongting Lake wetland is an important habitat and over-wintering area for East Asian migratory birds, and is located at 28°30'-30°20' N and 111°40'-113°40' E in the Northeastern part of Hunan Province, China. In 2007, an influenza virus A/environment/Dongting Lake/Hunan/3-9/07 (H10N8) was isolated from water from Dongting Lake wetland. The whole genome of the isolated virus was sequenced, the phylogenetic trees of each gene segment were generated, and the pathogenicity of the strain for mice and SPF White Leghorn Chickens was studied. To study further its potential pathogenicity for mammals, the virus was passaged in mouse lung, and the pathogenicity and corresponding amino acid variations of the mouse-lung-adapted virus from passages 2, 4 and 6 (P2, P4 and P6) were compared with those of wild-type virus (P0).

## Results

### Virus isolation and sequence comparisons

An H10N8 influenza A virus was isolated from water samples from Dongting Lake wetland, and named as A/environment/Dongting Lake/Hunan/3-9/2007 (H10N8) (environment/DT/Hunan/3-9/07). The whole genome of the isolated virus was sequenced to understand the genetic character of the virus.

BLAST analysis of the eight gene segments of environment/DT/Hunan/3-9/07 revealed the presence of an HA gene that was closely related to that of A/duck/Mongolia/149/03 (H10N5), with a nucleotide sequence identity of 96% and amino acid sequence identity of 97% (Table 1). The nucleotide and amino acid sequences of the NA gene of the H10N8 strain showed 97% and 98% homology, respectively, with those of strain A/duck/Spain/539/2006 (H6N8) (Table 1). The basic polymerase gene (PB2) was common to both A/mallard/Italy/37/02 (H5N3) and A/mallard/250/02 (H7N1), with a nucleotide sequence identity of 97%. However, the amino acid sequence of PB2 was closely related to that of A/mallard/Italy/3401/05 (H5N1) and A/mallard/Netherlands/12/00 (H7N3), with 99% identity (Table 1). The nucleotide sequence of the PB1 gene of the H10N8 strain showed 98% homology with that of the low-pathogenicity influenza virus strain A/duck/Denmark/65047/04(H5N2) isolated in Denmark in 2004, and the amino acid sequence showed 99% homology with that of A/turkey/Italy/1325/2005 (H5N2) and A/mallard/Netherlands/12/2000 (H7N3) (Table 1). The nucleotide and amino acid sequences of the PA gene of the H10N8 strain showed 97% and 99% homology, respectively, with those of the strain A/mallard/Italy/3401/2005 (H5N1) (Table 1). The nucleotide sequence of the NP gene of the H10N8 strain showed 98% homology with that of strain A/migratory duck/Jiang Xi/13487/2005 (H5N3), whereas the amino acid sequence showed 99% homology to that of strains A/Tree sparrow/Henan/4/2004 (H5N1) and A/duck/Jiang Xi/2374/2005 (H3N6) (Table 1). The matrix gene (M) of the H10N8 strain had 98% nucleotide sequence identity with A/duck/Hokkaido/Vac-2/04 (H7N7) and A/duck/Hokkaido/Vac-1/04 (H5N1). The amino acid sequence of the M1 gene had 100% identity with A/duck/Korea/S9/03 (H3N2) (Table 1). The nucleotide sequence of the non-structural gene (NS) of the H10N8 strain was most closely related to that of A/mallard/Yanchen/05 (H4N6) and A/duck/Jiangxi/1760/03 (H7N7), with 98% identity. The amino acid sequence of the NS1 gene of the H10N8 strain showed 98% identity with that of strains A/duck/Shantou/7488/2004 (H9N2) and A/mallard/Ohio/217/1998 (H6N8) (Table 1).

**Table 1:** Comparisons of A/environment/Dongting lake/Hunan/3-9/2007(H10N8) with isolates in GenBank of highest nucleotide and amino acid identity (%)^§^

Gene	Site	Nucleotide sequence Isolate with the highest homology	Homology (%)	Site	Amino acid sequence Isolate with the highest homology	Homology (%)
HA	20-1705	duck/Mongolia/149/03(H10N5)	96	1-561	mallard/Bavaria/3/06(H10N7)	97
					duck/Mongolia/149/03(H10N5)	97
NA	21-1433	duck/Spain/539/06(H6N8)	97	1-470	duck/Spain/539/06(H6N8)	98
		Mallard/65112/03(H3N8)	97		Mallard/65112/03(H3N8)	97
PB2	28-2307	mallard/Italy/37/02(H5N3)	97	1-759	mallard/Italy/3401/05(H5N1)	99
		mallard/Italy/250/02(H7N1)	97		mallard/Netherlands/12/00(H7N3)	99
PB1	25-2298	duck/Denmark/65047/04(H5N2)	98	1-757	turkey/Italy/1325/05(H5N2)	99
		turkey/Italy/3807/04(H7N3)	97		mallard/Netherlands/12/00(H7N3)	99
PA	22-2170	mallard/Italy/3401/05(H5N1)	97	1-716	mallard/Italy/3401/05(H5N1)	99
					duck/JiangXi/2374/05(H3N6)	99
NP	46-1527	migratory duck/JiangXi/13487/05(H5N3)	98	1-498	Tree sparrow/Henan/4/04(H5N1)	99
					duck/Jiang Xi/2374/05(H3N6)	99
M	1-1027	duck/Hokkaido/Vac-2/04(H7N7)	98	1-252	duck/Korea/S9/03(H3N2)^a^	100
		duck/Hokkaido/Vac-1/04(H5N1)	98			
NS	1-890	mallard/Yanchen/05(H4N6)	98	1-230	duck/Shantou/7488/04(H9N2)^b^	98
		duck/Jiangxi/1760/03(H7N7)	98		mallard/Ohio/217/98(H6N8)^b^	98

^§^Comparisons were performed by using the Blast search tool available from GenBank.

^a ^Amino acid sequence of M1 protein was compared.

^b ^Amino acid sequence of NS1 protein was compared.

### Phylogenic analysis

**Figure 1:**
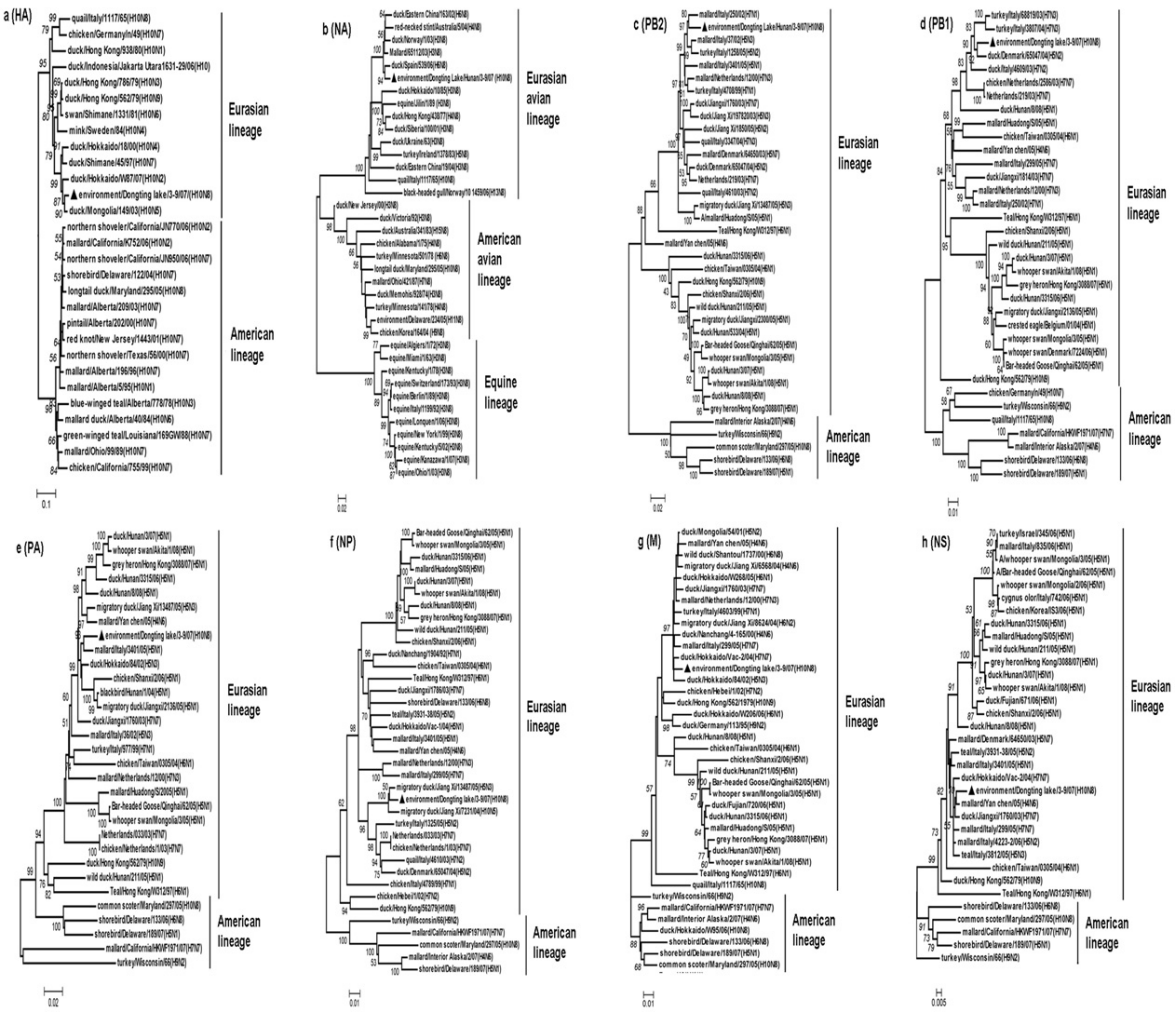
**Phylogenetic trees for the HA, NA, PB2, PB1, PA, NP, M and NS genes of the H10N8 influenza A virus**. Trees were generated by using neighbor-joining analysis with the Tamura-Nei model in the MEGA program (version 3.1). Numbers at the nodes indicate confidence levels of bootstrap analysis with 1000 replications as a percentage value. The scale bar represents the distance unit between the sequence pair.

Phylogenic analysis indicated that all the 8 gene segments of environment/DT/Hunan/3-9/07 were of aquatic avian origin and belonged to a Eurasian lineage. Phylogenic analysis of the HA gene revealed that it was closely related to Eurasian aquatic isolates (Figure 1a). The N8 NA genes of influenza A viruses were divided into 3 groups, namely, equine lineage, avian viruses isolated in the Eurasian region, and avian viruses isolated in North America [4]. The NA gene of environment/DT/Hunan/3-9/07 belonged to the lineage of avian viruses isolated in the Eurasian region (Figure 1b). The PB2 and PA genes of the H10N8 strain clustered together with the corresponding genes from H5 and H7 subtypes isolated from ducks and mallards in the Eurasian region (Figure 1c and 1e). However, the PB1 gene of the H10N8 strain formed a branch on the phylogenic tree together with those from H7 avian influenza viruses isolated from ducks, turkeys, and humans in some European countries, which indicated the same origin for these genes (Figure 1d). The NP gene of the isolated strain formed a relatively independent branch on the phylogenic tree, together with those from H5N3 and H10N5 viruses of Eurasian lineage (Figure 1f). M and NS genes of the isolated strain belonged to the Eurasian lineage too (Figure 1g and 1h).

### Chicken study

To determine the pathogenicity of environment/DT/Hunan/3-9/07, 8 SPF chickens were inoculated intravenously with virus in a volume of 0.2 ml (10^6.3^EID_50_), and another 8 chickens were inoculated intranasally with virus in a volume of 0.1 ml (10^6.0^EID_50_), and observed for clinical signs of disease and mortality for 14 days. The oropharyngeal and cloacal swabs of chickens were collected on days 3, 5 and 7 post inoculation (p.i.). for virus titration. None of the chickens challenged by intravenous or intranasal virus showed any clinical signs of disease within 14 days p.i., and none died during the observation period. These results suggested that the H10N8 strain was a low or non-pathogenic virus. Sera were harvested from the chickens at 21 days p.i. and seroconversion was confirmed by hemagglutination inhibition (HI) test. All the inoculated birds were seroconverted, although the HI antibody titers remained low throughout the experimental period (Table 2).

**Table 2:** Pathotyping and replication of the H10N8 virus in chickens^§^

Infection route	Days of post infection	Virus isolated from swabs				No.of Survivors	** No.of Seroconverted Chickens**^b^	** HI titers (Log**_**2**_**)**
					
		Oropharyngeal		Cloacal				
					
		No.of Chickens shedding virus	Titer^a^ (log_10 _EID_50_/ml)	No.of Chickens shedding virus	Titer^a^ (log_10 _EID_50_/ml)			
Intravenous (8)	3	3	1.7 ± 0.3	4	3.1 ± 0.7	8	8	6.3 ± 0.5
	5	7	3.8 ± 0.6	4	3.0 ± 0.7			
	7	5	1.7 ± 0.5	3	1.6 ± 0.5			
Intranasal(8)	3	5	2.3 ± 0.8	4	2.0 ± 0.3	8	8	5.6 ± 1.2
	5	8	3.4 ± 1.1	7	3.3 ± 0.1			
	7	6	2.5 ± 0.9	5	1.3 ± 0.4			

^§^One group of 8 six-week-old specific-pathogen-free white leghorn chickens were inoculated with 0.2 ml of 1:10diluted stock virus (10^6.3 ^EID_50_) intravenously and another group were inoculated with 10^6.0 ^EID_50 _of the virus in a 0.1 ml volume intranasally, and observed for 2 weeks after infection.

^a ^The mean titer in EID_50_/ml of swab media of the positive chickens.

^b ^Sera were harvested 3 weeks after infection, and seroconversion was confirmed by HI test.

### Mouse study

**Figure 2:**
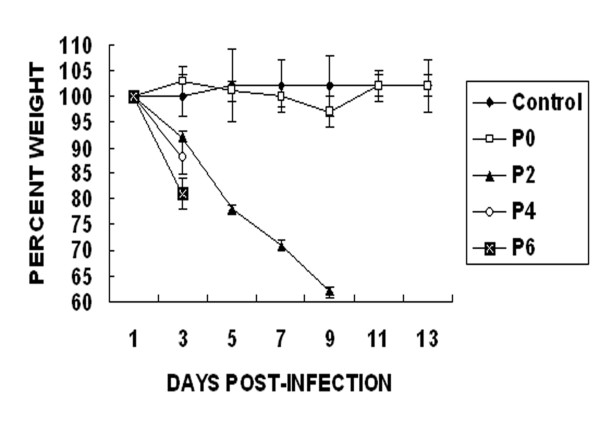
**Changes in body weight of BALB/c mice infected with different passages of the H10N8 virus**. Each mouse in a group was intranasally infected with 10^5.5^EID_50 _of the virus from different passage (P0, P2, P4 or P6) in a volume of 50 μl. The mice inoculated with lung washes prepared from uninfected mice served as a background control. The body weight of each mouse was expressed as the percentage of its weight on the day after infection. All the P2-infected mice died within 11 days after infection, whereas P4- and P6-infected mice died within 5 days.

Wild-type environment/DT/Hunan/3-9/07 showed no obvious pathogenicity towards BALB/c mice, and no obvious body weight loss was observed in inoculated mice (Figure 2), but high virus titers were detected in the lungs of mice on days 3 and 5 p.i. (Table 3). However, replication of wild-type virus was restricted in the lungs of mice, and no virus was recovered from other organs.

**Table 3:** Replication of the H10N8 virus from P0, P2, P4, P6 in mice^§^

Virus Strain	Days of post infection	**Virus titre [log**_**10**_**(EID**_**50**_**)] in**:				**MLD**_**50**_^**a **^**(log**_**10 **_**EID**_**50**_**)**
			
		brain	lung	spleen	kidney	
P0	3	-	3.7 ± 0.9	-	-	>6.5
	5	-	4.7 ± 1.5	-	-	
P2	3	1.4 ± 0.8	6.7 ± 0.4	+	1.6 ± 0.4	4.7
	5	+	6.3 ± 0.5	+	+	
P4	3	2.0 ± 0.3	6.8 ± 0.5	3.4 ± 0.5	2.4 ± 0.6	3.6
	5	ND	ND	ND	ND	
P6	3	2.4 ± 0.7	6.6 ± 0.4	3.6 ± 0.3	3.7 ± 0.3	3.2
	5	ND	ND	ND	ND	

^§ ^Six-week-old BALB/c mice were infected intranasal with 10^5.5^EID_50 _of the viruses from different passage (P0,P2,P4,P6). Organs were collected on days 3 and 5 after infection, and clarified homogenates were titrated for virus infectivity in 10-day-old SPF embryonated chicken eggs.

^a ^The MLD_50 _dose was determined by inoculating groups of five 6-week-old female mice intranasally with 10-fold serial dilutions of each virus according to Reed and Muench method.

- , Virus was not detected in the samples.

+, Virus was simply detected in undiluted samples.

ND, not done.

To evaluate further the potential pathogenicity of the H10N8 strain for mammals, the virus was subjected to lung-to-lung passage in mice. The virulence of environment/DT/Hunan/3-9/07 increased rapidly during adaptation in mouse lung. The result showed that, after two lung passages (P2), the virus caused fatal infection in mice. Mice inoculated with P2 virus showed serious clinical signs of disease such as ruffled fur, less movement and body weight loss (Figure 2), and viruses were recovered from multiple organs including the brain on days 3 and 5 p.i. (Table 3). Death of mice inoculated with P2 virus occurred on day 7 p.i., and all the 6 inoculated mice died within 11 days p.i. After 4-6 lung-to-lung passages, the virulence of the virus was enhanced further. The mice inoculated with P4 or P6 virus had the similar clinical signs of disease to those infected with P2 virus, but the mice inoculated with P4/P6 virus demonstrated more rapid and serious symptom onset compared with P2-infected mice (Table 3). The mice inoculated with P4 virus all died within 5 days p.i., whereas those inoculated with P6 virus all died within 4 days p.i. (Figure 2).

### Molecular changes during virus adaptation in mouse lung

To study further the molecular changes involved in the enhanced virulence of mouse-adapted virus, the whole genomes of P2, P4 and P6 viruses were sequenced, and their amino acid sequences were compared with those of wild-type virus strain (P0).

It was found that, during passage in the mouse lung from P0 to P6, 22 amino acid substitutions appeared, i.e. sites 207, 616 and 627 of PB2 gene; sites 247 and 611 of PA gene; sites 94, 244, 252, 386 and 430 of HA gene; site 479 of NP gene; sites 21, 32, 286, 330 and 385 of NA gene; sites 53 and 192 of M1 gene; site 82 of M2 gene; and sites 54, 89 and 155 of NS1 gene (Table 4). No amino acid substitutions were observed in PB1 or NS2 genes during passage in murine lung from P0 to P6 (Table 4).

**Table 4:** Amino acid sequence comparison of virus from P0, P2, P4, P6^§^

Gene	Amino acid position	Amino acid in virus			
		
		P0	P2	P4	P6
PB2	207	L	V	V	V
	616	V	I	I	I
	627	E	E	K	K
PB1	-	-	-	-	-
PA	247	S	S	S	A
	611	F	F	F	S
HA	94	P	L	L	L
	244	R	W	W	W
	252	N	N	N	H
	386	V	V	D	D
	430	Y	Y	D	D
NP	479	L	F	F	F
NA	21	I	N	N	N
	32	A	T	T	T
	286	V	V	A	A
	330	Q	Q	Q	R
	385	K	K	R	R
M1	53	S	S	S	P
	192	M	M	M	V
M2	82	S	S	S	G
NS1	54	T	I	I	I
	89	Y	Y	Y	H
	155	A	A	A	V
NS2	-	-	-	-	-

^§^The whole genome of the viruses from P0, P2, P4, P6 were sequenced, and the amino acid sequences of the corresponding gene segments was aligned.

-, No amino acid substitution was found.

## Discussion

Among all 16 HA and 9 NA subtypes of influenza A viruses, the highly pathogenic avian influenza viruses are restricted to subtypes H5 and H7, although not all H5 and H7 viruses are virulent. However, low-pathogenicity viruses previously have been shown to be precursors of highly pathogenic viruses [5,6]. The H10N8 strain isolated in the present study replicated efficiently in mouse lung without prior adaptation. Its pathogenicity for mice increased rapidly during lung adaptation, and even after 2 passages, it became lethal for mice. It has been reported that H11N9 subtype virus can be transmitted directly from wild ducks to waterfowl hunters[7]. Therefore, when emphasis is placed on H5, H7 and H9 subtype avian influenza viruses, the other subtypes should not be ignored, because they might also be a potential threat to public health.

Migratory birds that carry avian influenza virus might shed virus into the environment along their migratory route. After the birds leave an area, environmental persistence of the virus could play an important ecological role in virus transmission [8,9]. Shedding of the virus into water could lead to infection of any waterfowl that are dabbling in the same area, via the direct or indirect fecal-oral route[2]. Animals that utilize an area in which viruses persist might experience increased viral exposure, and therefore, greater potential for viral infection and reassortment [8].

Phylogenic analysis showed that all the gene segments of environment/DT/Hunan/3-9/07 belonged to the Eurasian lineage, but some gene segment of the virus had different origin. It is believed that all 16 subtypes of HA and 9 subtypes of NA are perpetuated in the aquatic bird population, and reassorted with each other with a high frequency [1,2]. It is assumed that, when viruses of different origin are mixed somewhere in the habitats or aggregation sites along the migration route, gene reassortment takes place [10]. The virus strain isolated in the present study could have been resulted from multiple gene segments reassortment between different viruses, including H5 and H7 subtypes.

The virus strain isolated in this study replicated effectively in mouse lung without prior adaptation. During adaptation, the virus demonstrated extrapulmonary spread and enhanced replication in the mouse, and the viruses were recovered from multiple organs, including the brain. The virulence of the strain in mice increased rapidly and became lethal after only 2 lung-to-lung passages. The host specificity and pathogenicity of influenza A virus have always been considered as being determined by multiple genes [11,12]. However, the genetic basis for virulence of influenza A virus is largely unknown [13]. During 6 passages of the H10N8 strain in mouse lung, amino acid substitutions were observed at 22 sites in the viral genome (Table 4). These demonstrated that multiple amino acid substitutions were likely to have been involved in the adaptation of the virus to mice. It has been reported that the amino acid substitution from E to K at site 627 of the PB2 gene is the first step in virus adaptation in mammals, and that this substitution is host-dependent [14,15]. Therefore, we deduced that the PB2-E627K substitution significantly enhanced the pathogenicity of the H10N8 strain for mice. However, after 2 lung-to-lung passages, viral pathogenicity was also enhanced and caused death, compared with the wild-type virus, but there was no amino acid substitution at the 627 site in the PB2 gene of P2 virus, which indicated that the amino acid substitutions at other sites in the viral genome were also involved in the increased virulence of mouse-lung-adapted virus strains. It has also been shown that molecular changes at specific sites of PA and PB1 genes are associated with high pathogenicity of the H5N1 virus [16]. However, no amino acid substitution was observed in PB1 gene during virus adaptation, whereas the amino acids 247 and 611 of PA were substituted. The amino acid at site 479 of the NP gene of the virus strain isolated in the present study was substituted from L to F during passage in murine lung, which might influence NP oligomerization [13,17]. The activity-enhancing mutations of the viral polymerase complex that consists of PB2, PB1, PA and NP might be a prerequisite for adaptation to a new host [17,18].

The amino acids at 5 sites of the HA gene were substituted during passage of the virus in mouse lung. In the H5N1 subtype viruses, the multiple basic acids adjacent to the cleavage site of the HA gene are a prerequisite for lethality in mice and chickens [19]. The pathogenicity of the H10N8 virus isolated in this study increased rapidly during passage in mouse lung, although no amino acid substitutions were observed near the cleavage sites of its HA gene. The balance between neuraminidase activity of the NA gene and receptor-binding activity of the HA gene is closely associated with replication of influenza virus in the host [20]. Studies have shown that M1 gene mutation during passage in mouse lung might enhance virus replication, which results in enhanced pathogenicity [21]. The amino acid substitutions at sites 53 and 192 of the M1 gene might have close relationship with viral pathogenicity. NS1 protein plays an important role in counteracting the host interferon system [22], and is closely related to viral pathogenicity and host specificity [23,24]. In the present study, the amino acids at sites 54, 89 and 155 of the NS1 gene were substituted. It should be noticed that the substitution from Y to H at site 89 might be closely related to pathogenicity and adaptation of influenza A virus, because the same mutation has been observed at the same site during H9N2 virus adaptation in mouse lung [12]. Amino acid substitutions were observed at multiple sites of the genomes of the H10N8 strain during adaptation in mouse lung. Comparison of the genomic amino acid sequence of P0, P2, P4 and P6 viruses are helpful in understanding the molecular mechanism of pathogenicity of influenza A virus.

When the virus was passaged in the mouse lung from P0 to P6, 22 amino acid substitutions appeared. Some of these substitutions might be introduced randomly and maintained, whereas others are selected during adaptation of the virus in mice. Some substitutions such as the PB2-E627K, NP-L479F and NS1-Y89H have been found during the other influenza virus adaptation in mouse lung [12,13,17]. However, whether these amino acid substitutions lead to increased virus virulence in chickens remains unknown. The wild-type H10N8 strain showed no significant pathogenicity towards SPF chickens, but the infected chickens had shed virus through the respiratory tract and cloaca. The H10N8 virus isolated in present study possesses internal genes of both H5 and H7 subtype origin, which might provide gene segments for further gene reassortment between various influenza A viruses. It is assumed that the wider the circulation of low-pathogenicity avian influenza virus in poultry, the higher the chance that mutation to high-pathogenicity virus will occur [6]. Low-pathogenicity viruses previously have been shown to be the precursors of high-pathogenicity viruses [5,6].If such a virus is allowed to circulate in poultry or wild birds, mutations may merge, and the low-pathogenicity virus could become more pathogenic by gene mutation or reassortment.

Influenza A viruses have been maintained in waterfowl populations by water-borne transmission [25]. Shedding of the virus into the water is a major threat for epidemics in poultry [2]. Therefore, water persistence of viruses might play an important ecological role in virus transmission. Monitoring the water at aggregation and breeding sites of migratory waterfowl, mainly wetland, is very important for early detection of avian influenza virus [3]. Dongting Lake wetland is an important habitat and overwintering area along the migration route of migratory birds in East Asia. In the wetland, domestic ducks often share with wild waterfowl the same water area for dabbling and habitat, which provides ample opportunity for influenza virus to infect domestic ducks and other domestic poultry. Thus, investigation of water in Donting Lake wetland for avian influenza virus is of greater significance and convenience for understanding the route and mechanism of virus transmission between domestic fowl and migratory birds.

## Conclusions

In the wetland, water persistence of viruses might play an important ecological role in virus transmission. The avian influenza viruses might be transmitted among wild and domestic waterfowls through waterway. It should be noted that the H10N8 subtypes of avian influenza viruses might evolve to pose a potential threat to mammals and multiple amino acid substitutions are likely to be involved in the adaptation of H10N8 influenza virus to mice.

## Materials and methods

### Ethics Statement

Specific-pathogen-free (SPF) BALB/c mice (females, aged 6-8 weeks old) were purchased from Hubei Research Center of Laboratory Animal, China. The SPF white Leghorn chickens (aged 6 weeks old) were purchased from Beijing Merial Vital Laboratory Animal Technology CO., LTD. Mice and Chickens were all bred in the Animal Resource Center at the Wuhan Institute of Virology, Chinese Academy of Sciences, maintained in specific-pathogen-free conditions prior to infection, and cared for under MOST (Ministry of Science and Technology of the People's Republic of China) guidelines for laboratory animals. All experiments involved in animals have been approved by Animal Care Committee of Wuhan Institute of Virology, Chinese Academy of Sciences.

### Sample collection

In October 2007, 95 water samples from areas near the habitat of migratory birds in East Dongting Lake, Yueyang City, Hunan Province were collected by using sterilized 200-ml screw-cap plastic vials. A 200-ml water sample was collected at each sampling site, stored in a portable refrigerator, sent to our laboratory, and stored at -80°C until assayed.

### Virus isolation and purification

Seventy milliliters of each water sample was transferred into a sterilized 80-ml polyethylene plastic tube with a screw-cap and round bottom, under aseptic conditions. Polyethylene glycol 6000, sodium chloride and bovine serum albumin (BSA) were added to final concentrations of 8%, 3% and 0.1%, respectively, mixed gently, set on ice for 8-12 h during which the tube was inverted every 2 h to mix the contents, and centrifuged at 4°C, 10,000×*g *for 30 min[26]. The supernatant was discarded, and the precipitate was re-suspended in 1 ml PBS, which contained 2 × 10^6 ^U/l penicillin, 2 × 10^6 ^U/l amphotericin B, 250 mg/l kitasamycin, 0.5 × 10^6 ^U/l nystatin, and 60 mg/l ofloxacin HCl. Then, 0.5 ml of the re-suspended mixture was inoculated into the allantoic cavities of 10-day-old specific-pathogen-free (SPF) embryonated chicken eggs and incubated at 37°C for 72 h. The allantoic fluid with hemagglutination titers were harvested and confirmed as influenza A virus stock by RT-PCR, using NP-gene-specific primers and universal primers for the M gene of influenza A virus, as described previously[27,28]. The confirmed influenza virus stock was aliquoted and stored at -80°C before use.

The viruses were clonally purified by plaque isolation in MDCK monolayers, followed by stock preparation as described previously [11,13].

### Genetic and phylogenic analysis

Total RNA from the virus genome was extracted from the prepared virus stock by lysing with Trizol LS reagent (Life Technologies) and reverse-transcribed into single-stranded DNA with M-MuLV reverse transcriptase (New England Biolabs). All segments were amplified with Phusion™ High-Fidelity PCR Kit (New England Biolabs). The PCR products were purified with the Cycle-pure Kit and Gel Extraction Kit (OMEGA), and the fragments were cloned into pGEM-T easy vector and sequenced by the dideoxy method with an ABI 3730 DNA sequencer (Applied Biosystems). Three clones of each gene were selected for repeated sequencing to confirm that the sequence data obtained on the two occasions were identical[29]. Data were edited and aligned by BioEdit version 7.0.5.2.

Phylogenic trees were generated with neighbor-joining bootstrap analysis (1000 replicates) using the Tamura-Nei algorithm in MEGA version 3.1 [30].

### Chicken study

Eight SPF White Leghorn Chickens aged 6 weeks were intravenously inoculated with 0.2 ml of a 1:10 dilution of bacteria-free allantoic fluid that contained virus (10^6.3 ^EID_50_). Meanwhile, another 8 chickens aged 6 weeks were inoculated intranasally with 0.1 ml 10^6.0 ^EID_50 _virus. The inoculated chickens were observed for 14 days for mortality and clinical signs of disease. Tracheal and cloacal swabs were collected on days 3, 5 and 7 post inoculation (p.i.) for virus titration[31]. The EID_50 _was calculated by the Reed and Muench method. Sera were harvested from the inoculated chickens on day 21 p.i. for seroconversion confirmation by hemagglutination inhibition (HI) assays with 0.5% chicken erythrocytes according to the recommendation of OIE.

### Adaptation of the H10N8 strain in the mouse lung

Adaptation of the H10N8 strain in mouse lung was carried out by serial lung-to-lung passage, as described previously [11,32]. Ten female BALB/c mice aged 6 weeks were anesthetized and inoculated intranasally with 10^6.5^EID_50 _purified virus in a volume of 50 μl, and labeled as P0. The mice were sacrificed on day 3 p.i., and their lungs and trachea were taken out and washed 3 times with a total of 2 ml PBS that contained 0.1% BSA and antibiotics, as described previously [33]. The lung washes were centrifuged at 4°C, 4,000×*g *for 10 min, and the supernatant was harvested, aliquoted, and stored at -80°C, and labeled as P1[34]. The lung-to-lung passage tests were repeated 6 times, and labeled up to P6.

The BALB/c mice aged 6 weeks were divided into 4 groups of 16 each, anesthetized, and inoculated intranasally with P0 (wild-type), P2, P4 and P6 virus in a volume of 50 μl (10^5.5 ^EID_50_). Five mice in each group were dissected on days 3 and 5 p.i., and their lungs, spleens, kidneys and brains were taken out under aseptic conditions, weighed and homogenized with 1 ml PBS that had been pre-cooled in ice. Tissue homogenates were centrifuged at 4°C,4,000×*g *for 10 min to remove any tissue fragments, and used to determine virus titer[31]. The remaining 6 mice in each group were observed daily for weight loss and mortality. The 50% mouse lethal dose (MLD_50_) of the virus was determined by inoculating intranasally 5 groups of mice (*n *= 5 mice each) with 10-fold serial dilutions of the virus in a volume of 50 μl. The MLD_50 _was calculated by the method of Reed and Muench.

### Sequencing of the genomes of P2, P4 and P6 viruses

Total RNA was directly extracted from the lung washes of P2, P4 and P6 viruses as described previously [34]. The whole genomes of P2, P4 and P6 viruses were sequenced as described in the section of "Genetic and phylogenic analysis".

### Nucleotide sequence accession numbers

The nucleotide sequences for the viral genome of environment/DT/Hunan/3-9/07(P0) have been submitted to GenBank and are available under accession numbers GQ290464--GQ290471. The nucleotide sequences for the genomes of P2, P4 and P6 viruses are available under GenBank accession numbers GQ325634-GQ325657.

## Competing interests

The authors declare that they have no competing interests.

## Authors' contributions

HBZ carried out most of the experiments and wrote the manuscript. BX, QJC and JJC did part of the experiment. ZC was the main designer of the experiment and revised the manuscript. All authors read and approved the final manuscript.
